# The Proposed Central Hospitals Board for London

**Published:** 1893-04-29

**Authors:** 


### HOSPITAL OPINION.

[Contributions critical, suggestive, controversial, and technical are invited for this column, which is open to everyone who has anythinigto say that is of practicalvalue and interest.]

### THE PROPOSED CENTRAL HOSPITALS BOARD FOR LONDON.

A paragraph, which appears in your issue of 22nd inst., under the heading of "Notes and News," states that "a minority of the hospital secretaries are, we understand, actively fermenting a strenuous opposition to the proposed Central Hospitals Board. It is reported that a meeting of these gentlemen has been held at the Royal Free Hospital."

This paragraph is so entirely incorrect and misleading that I desire to correct it by a statement of the facts respecting the meeting referred to.

Being desirous of ascertaining what were the opinions of some of my brother officials on the subject of the proposed Central Board, I invited about thirty hospital secretaries to a social gathering, for the purpose of affording an opportunity for an informal conversation on this question, which is of paramount importance to all who are engaged in hospital work. It was distinctly understood that we met solely for interchange of views, and that no resolutions would be brought forward. Whatever may have been the opinions expressed by others, I may venture to state that, speaking as a private individual and not as representing any institution, I am personally not averse to some kind of central board, although I am not able to approve the schemes which have already been brought forward. I am, yours faithfully,

Conrad W. Thies.

Royal Free Hospital, Gray's Inn Road, W.C.
April 24th, 1893.

*** We publish this letter with pleasure, although it does not controvert in any way the statement that "a minority of the hospital secretaries are fermenting a strenuous opposition to the proposed Hospitals Board." It is important that this statement should be definitely confirmed or refuted. Besides, Mr. Thies only states his own views, without in any way denying that the majority of the speakers at the meeting in question were hostile to the formation of a Central Hospitals Board for London.—Ed. *T. H.*

